# Adverse Drug Reactions of Low and Standard Doses of Risperidone in Schizophrenia Outpatients

**DOI:** 10.2174/0115748863369843250327054632

**Published:** 2025-04-18

**Authors:** Tuanthon Boonlue, Praewrawee Karnkla, Sadanan Jansela, Chompoonuch Werawattanachai

**Affiliations:** 1 Faculty of Pharmaceutical Sciences, Ubon Ratchathani University, Ubon Ratchathani, Thailand;; 2 Department of Pharmacy, Prasrimahabhodi Psychiatric Hospital, Ubon Ratchathani, Thailand

**Keywords:** Adverse drug reactions, antipsychotic drugs, outpatients, Gastrointestinal system, risperidone, schizophrenia, dystonia

## Abstract

**Introduction:**

Risperidone is an atypical antipsychotic commonly used in the treatment of schizophrenia. Despite its effectiveness, several adverse drug reactions can be bothersome to patients. This study aimed to examine the prevalence and characteristics of adverse effects in outpatients with schizophrenia treated with low (<4 mg/day) and standard (≥4 mg/day) doses of risperidone.

**Methods:**

A cross-sectional study was conducted with 64 patients at a tertiary psychiatric hospital. Data on adverse effects were collected through a self-report questionnaire, and causality was assessed using Naranjo’s Algorithm. Descriptive statistics and chi-square tests were employed for data analysis.

**Results:**

The participants comprised 51.56% females, with a mean age of 45.16±14.32 years. Significant gender differences were observed between dose groups, with more females in the low-dose group (63.16%) than in the standard-dose group (34.62%) (*P* = 0.02). A total of 221 adverse effects were confirmed after assessment. The most common effects were weight gain (57.81%), increased appetite (48.44%), and dystonia (32.81%). Weight gain was more prevalent in the low-dose group (68.42%) than the standard-dose group (42.31%; Cohen's h = 0.53, 95% CI: 2.0%–50.2%), while insomnia was higher in the standard-dose group (23.08%) compared to the low-dose group (5.26%; Cohen's h = 0.54, 95% CI: 0.1%–35.5%).

**Discussion:**

These findings highlight the importance of monitoring dose-dependent ADR patterns and tailoring interventions to individual patient needs. Several limitations should be acknowledged, including the small sample size and the cross-sectional design, which limits causal inference.

**Conclusion:**

Weight gain was the most prevalent adverse effect associated with risperidone use, particularly at lower doses, while insomnia was more frequent at standard doses, emphasizing the need for careful monitoring and personalized dose adjustments to optimize patient safety.

## 1 INTRODUCTION

Risperidone is an atypical antipsychotic used for the treatment of schizophrenia, offering better acceptance among patients compared to typical antipsychotics [1]. A study on relapse rates in outpatients with clinically stable schizophrenia or schizoaffective disorder treated with either haloperidol or risperidone found that those in the risperidone group had significantly lower relapse rates [2]. In addition, evidence suggests that maintenance treatment with antipsychotic drugs for individuals with schizophrenia offers several other benefits. It not only helps prevent relapses and rehospitalizations but also improves the overall functioning and quality of life of the patient, and supports sustained remission [3]. However, this benefit must be carefully balanced against the potential Adverse Drug Reactions (ADRs) associated with long-term antipsychotic use [4].

The effective dose range for risperidone is 4–6 mg/day for the acute phase, while a reduced dose of 3–4 mg/day is typically recommended during the maintenance phase, which aims to minimize dose-dependent side effects, thereby improving patient safety and tolerability over the long term [5, 6]. However, meta-analyses have suggested that in the maintenance phase of multi-episode schizophrenia, reducing antipsychotic doses below the standard range recommended for acute stabilization may not be advisable, as this is associated with a higher risk of relapse and all-cause treatment discontinuation [7]. Thus, balancing the risk benefits of antipsychotics in the maintenance phase is recommended, and clinicians should take several risk factors for psychotic relapse into account when considering dose reduction in patients with chronic schizophrenia [8].

The dose of antipsychotic during maintenance based on the Defined Daily Dose (DDD) of the World Health Organization can be categorized as follows: standard doses refer to ≥1 DDD, low doses range from ≥50% to <1 DDD, and very low doses are defined as <50% DDD.Meta-analyses have shown that low-dose therapy does not significantly differ from standard-dose treatment in terms of overall treatment failure or hospitalization rates, and no significant differences were observed in the dropout rates due to side effects between standard and either low or very low doses [9].

Risperidone has a high affinity for 5HT2A and 5HT7 receptors while also demonstrating strong binding to dopamine D2 receptors, as well as α1 and α2 adrenergic receptors [10]. Risperidone tends to cause slightly more extrapyramidal side effects and significantly higher prolactin levels compared to most other second-generation antipsychotics. It may also differ from others in its efficacy and the occurrence of other adverse effects, including weight gain, metabolic disturbances, cardiac effects, sedation, and the risk of seizures [11]. Understanding the prevalence and nature of these ADRs is critical, particularly during the maintenance phase of treatment, where prolonged use may increase the risk of side effects [4].

While it is well recognized that higher doses of risperidone may increase the risk of ADRs [12], the extent to which dose-dependent variations in ADRs manifest in real- world clinical settings remains underexplored. Previous research has often focused on controlled clinical trials, which may not fully reflect the complexities of routine outpatient care, such as demographic variability, co-morbidities, and concomitant medications [13-16]. Understanding dose-dependent ADRs in real-world practice is critical for optimizing treatment strategies, as it allows clinicians to weigh therapeutic efficacy against the risk of adverse effects and personalize treatment accordingly.

This study aims to address these gaps by comparing the prevalence and characteristics of ADRs between low (<4 mg/day) and standard (≥4 mg/day) doses of risperidone among outpatients diagnosed with schizophrenia during the maintenance phase of treatment.

## 2 METHODS

### 2.1 Study Design

This study was a cross-sectional descriptive study aimed at monitoring ADRs of low *vs* standard doses of risperidone in outpatients with schizophrenia. Data collection was performed using a structured, self-administered questionnaire designed to gather information on adverse effects, demographics, and treatment-related factors.

### 2.2 Study Setting

Participants were recruited from A tertiary Psychiatric Hospital's outpatient clinic between July and December 2018. Eligible patients were identified through electronic health records, and recruitment was conducted during routine clinic visits. Written informed consent was obtained from all participants. However, as this study was conducted at a single center, the findings may have been influenced by the specific patient population and clinical practices of the institution, which could limit the generalizability of the results to other settings.

### 2.3 Sample Size

The sample size for this study was determined based on data from a previous study that examined the safety and tolerability of four risperidone dose levels (2, 6, 10, and 16 mg) in patients with schizophrenia [17]. In that study, the estimated prevalence of adverse effects in the low-dose group (2 mg) was approximately 25%, while the prevalence in the standard-dose group (6 mg or higher) was around 50%. To detect a significant difference between the two groups with a two-sided significance level of 5% and a power of 80%, the required total sample size was calculated to be 55 patients.

### 2.4 Participants Selection Criteria

The study included male and female outpatients aged 18 years or older, diagnosed with schizophrenia under ICD-10 codes F20-F29. Eligible participants were those who had been prescribed and consistently taken risperidone, with self-reported adherence of more than 80% for at least two weeks prior to the assessment of ADRs. Only patients with stable psychiatric symptoms, as evaluated and documented by a psychiatrist in their medical records, were included.

Exclusion criteria were as follows: patients who were concurrently receiving other antipsychotic medications, except for those on low-dose chlorpromazine (<100 mg/day), which is commonly used for treating insomnia in our setting; individuals unable to read or write, thus preventing completion of the study questionnaire, and patients with incomplete data or missing essential information required for analysis.

### 2.5 Validation of Instruments

The questionnaire was developed based on a comprehensive review of literature related to risperidone-induced ADRs [14, 18-20]. Content validity was assessed using the Index of Item Objective Congruence (IOC), where three independent experts (one psychiatrist and two clinical pharmacists) evaluated the relevance and appropriateness of each item. Items with an IOC score of 0.75 or higher were retained, resulting in a final IOC score of 0.86, which indicated high content validity.

To ensure data quality and completeness, several measures were implemented. First, the questionnaire underwent pilot testing with 10 eligible patients to evaluate its clarity and appropriateness. Feedback from the pilot test was incorporated into the final version of the questionnaire. Second, research assistants received training to provide standardized instructions to participants, reducing variability and minimizing the risk of bias. Third, completed questionnaires were reviewed in real-time during clinic visits to identify any missing or inconsistent responses. Participants were asked to clarify or complete the information immediately, ensuring data accuracy and completeness. Lastly, participants were assured of anonymity and confidentiality, encouraging honest and thorough responses. These measures collectively enhanced the reliability and validity of the data collected for the study.

### 2.6 Variables

The primary variables included ADRs, patient demographics, and treatment-related factors. Data on ADRs were collected using a structured, self-administered questionnaire developed by the researchers. Patients completed the questionnaire during their clinic visit. Naranjo’s algorithm was applied by a trained pharmacist to assess the likelihood of a causal relationship between reported ADRs and risperidone use.

Patient demographics collected included age, gender, and psychiatric history. Treatment-related factors encompassed the dose of risperidone, categorized as low dose (< 4 mg/day) and standard dose (≥ 4 mg/day)21, and the duration of treatment. The aim of this study was to compare the prevalence and characteristics of ADRs between these two dosing groups.

The study also considered clinical characteristics, such as the duration of schizophrenia and stability of psychiatric symptoms, to provide context for interpreting ADRs. Continuous variables (*e.g*., age, duration of treatment) were recorded as means and standard deviations, while categorical variables (*e.g.,* gender, type of ADR, dose group) were reported as frequencies and percentages.

### 2.7 Statistical Analysis

Descriptive statistics were used to summarize baseline characteristics. Continuous variables are reported as mean ± standard deviation (SD), while categorical variables are presented as frequencies and percentages. Differences in ADR prevalence between dose groups were assessed using chi-square tests or Fisher’s exact tests, where appropriate. Continuous variables were analyzed using t-tests or Mann-Whitney U tests, depending on the distribution. Statistical significance was set at *P* < 0.05, and confidence intervals were reported for significant findings.

### 2.8 Ethics Approval

This study was approved by the Institutional Review Board (IRB) of Prasrimahabhodi Psychiatric Hospital with the approval reference number [COA No. 007/2561]. All participants provided written informed consent before participating in the study. Data confidentiality was maintained throughout the study, and participants were assured that their responses would be anonymized.

## 3 RESULTS

### 3.1 Baseline Characteristics

As shown in Table **1**, the study included 64 patients distributed across two dose groups, with 38 patients in the low-dose risperidone group and 26 in the standard-dose group. There was a significant difference in gender distribution, with a higher proportion of females in the low-dose group (63.16%) compared to the standard-dose group (34.62%) (*P*= 0.02). The mean age of participants was 45.16 ± 14.32 years, with no significant difference between the two dose groups. Regarding marital status, 55.26% of patients in the low-dose group were single, compared to 76.92% in the standard-dose group (*P*= 0.08). Duration of risperidone treatment also showed no significant difference between the two groups, with a mean duration of 28.57 ± 24.96 months in the low-dose group and 33.5 ± 34.49 months in the standard-dose group (*P*=0.51). Although the diagnosis distribution was similar, unspecified nonorganic psychosis was more prevalent in the low-dose group (21.05%) than in the standard-dose group (3.85%) (*P*= 0.15). The median risperidone dose was significantly higher in the standard-dose group (4.80 mg) than in the low-dose group (1.72 mg) (P < 0.01). There was no significant difference in the history of drug allergies between the groups (*P*= 0.69).

Regarding psychiatric diagnoses, most participants in both groups were diagnosed with unspecified schizophrenia (ICD-10 code F20.9), accounting for 57.89% in the low-dose group and 73.08% in the standard-dose group. Paranoid schizophrenia (F20.0) was diagnosed in 21.05% of the low-dose group and 23.08% of the standard-dose group. A smaller proportion of participants were diagnosed with unspecified nonorganic psychosis (F29.0), representing 21.05% of the low-dose group and 3.85% of the standard-dose group. The distribution of psychiatric diagnoses did not significantly differ between the two groups (*p*= 0.15).

In terms of marital status, 76.92% of patients in the standard-dose group were single, compared to 55.26% in the low-dose group. Conversely, 44.74% of the low-dose group were married, compared to 23.08% in the standard-dose group. However, this difference was not statistically significant (*p*= 0.08). Most patients had no history of drug allergy, with 94.74% in the low-dose group and 92.31% in the standard-dose group, and there was no significant difference between the two groups (*p*= 0.69).

### 3.2 Adverse Drug Reactions Assessed by Naranjo’s Algorithm

The prevalence of ADRs was assessed in a cohort of 64 patients receiving risperidone, with both initial and final prevalence rates documented after applying Naranjo’s algorithm to determine causality shown in Table **2**. Among extrapyramidal symptoms, the initial prevalence of dystonia was 32.81%, which slightly decreased to 29.69% after the final assessment. Similarly, the initial and final prevalence of akathisia remained consistent at 9.38%, while parkinsonism also showed no change, with a prevalence of 20.31%. Tardive dyskinesia was observed in 14.06% of patients, consistent across both initial and final assessments. These findings indicate a stable occurrence of most extrapyramidal symptoms, except for a slight reduction in dystonia following the evaluation.

For central nervous system-related ADRs, headache and insomnia showed consistent prevalence rates of 10.94% and 12.5%, respectively, in both initial and final evaluations. Sedation was initially reported in 20.31% of patients but reduced to 17.19% after assessment. Dry mouth and weight gain were prominent ADRs, with dry mouth remaining at 31.25% and weight gain showing the highest prevalence at 57.81%, consistent across both assessments. Increased appetite and constipation were the primary gastrointestinal-related ADRs, reported in 48.44% and 26.56% of patients, respectively, with no changes between initial and final assessments.

In the category of endocrine and reproductive system ADRs, irregular menstruation was observed in 15.63% of patients, and loss of libido was noted in 4.69%, with both rates consistent across assessments. Cardiovascular-related ADRs included orthostatic hypotension (17.19%) and palpitations (14.06%), with no significant changes after applying Naranjo’s algorithm. Other reported ADRs, such as a rash (3.13%) and nasal congestion (10.94%), also remained stable across initial and final evaluations. Overall, the total number of ADR occurrences slightly reduced from 225 to 221 after applying causality assessment, highlighting the importance of verifying the association between symptoms and medication use.

### 3.3 Comparison of Adverse Drug Reactions between Low and Standard Dose Risperidone

The comparison of ADRs between the low-dose and standard-dose risperidone groups is presented in Table **3**. Among extrapyramidal symptoms, dystonia was more prevalent in the standard-dose group than the low-dose group (*P*= 0.20), while akathisia was more frequent in the low-dose group compared to the standard-dose group (*P*= 0.39). Parkinsonism and tardive dyskinesia occurred at comparable rates between groups, with no statistically significant differences (*P*= 0.86 and *P*= 0.29, respectively).

For central nervous system-related ADRs, weight gain and insomnia demonstrated significant differences between the dose groups. Weight gain was more prevalent in the low-dose group (68.42%) compared to the standard-dose group (42.31%), with a medium effect size (Cohen's h = 0.53, 95% CI: 2.0%–50.2%, *P*= 0.04). Conversely, insomnia was significantly more frequent in the standard-dose group (23.08%) compared to the low-dose group (5.26%), also with a medium effect size (Cohen's h = 0.54, 95% CI: 0.1%–35.5%, *P*= 0.03). Other CNS-related ADRs, such as headache, sedation, and dry mouth, did not differ significantly between the groups (*P*= 0.23, 1.00, and 0.63, respectively).

Gastrointestinal ADRs, such as increased appetite and constipation, were more common in the low-dose group than the standard-dose group. However, neither difference was statistically significant (*P*= 0.42 and *P*= 0.15, respectively). Endocrine and reproductive system ADRs (galactorrhea, irregular menstruation, and loss of libido) and cardiovascular ADRs (orthostatic hypotension and palpitations) occurred at low and comparable rates between groups, with no significant differences. Similarly, other ADRs, including rash and nasal congestion, showed no significant variation between groups. Overall, only insomnia and weight gain demonstrated statistically significant differences between the low- and standard-dose risperidone groups.

## 4 DISCUSSION

This study compared ADRs between low-dose and standard-dose risperidone groups among outpatients with schizophrenia. The analysis included 64 patients, with 38 in the low-dose group and 26 in the standard-dose group. Our findings provide insights into the patterns of ADRs associated with different dosing regimens and underline the importance of careful dose adjustment in clinical practice.

Our results revealed a significant difference in gender distribution between the groups, with a higher proportion of females receiving low-dose risperidone (63.16%) compared to the standard-dose group (34.62%) (*P*= 0.02). This may suggest a gender-based difference in medication tolerance or dosing preferences, potentially due to the increased sensitivity to antipsychotics observed in females, which has been reported in previous studies [21-23]. The review identified that women may have an increased susceptibility to weight gain, diabetes, and specific cardiovascular risks associated with antipsychotic use [24]. These effects are thought to result from slower drug absorption, metabolism, and excretion in women, leading to higher plasma drug concentrations, which in turn increase the risk of side effects. Additionally, women exhibit higher dopamine receptor occupancy compared to men at similar serum drug levels, potentially due to the influence of estrogens, which enhance dopamine sensitivity [22]. In terms of efficacy, the meta-analysis study found evidence suggesting that women exhibit a higher response to antipsychotic medication compared to men in the treatment of schizophrenia [25]. Thus, the management of schizophrenia with risperidone underscores the importance of gender-based differences in personalized treatment, with significant implications for both efficacy and safety considerations.

The sociodemographic characteristics observed in this study align with findings from similar populations. A previous study [26] comparing the safety and efficacy of chlorpromazine and risperidone also reported that treatment outcomes and adverse reactions were influenced by factors such as gender and age among patients with schizophrenia. Additionally, our study found no significant differences in marital status or treatment duration between the dose groups. Nevertheless, these factors remain important considerations for treatment adherence and the comprehensive management of schizophrenia [27, 28].

Our findings are in line with prior studies suggesting that the dose of antipsychotics, particularly risperidone, can influence the spectrum of ADRs observed. The overall assessment of ADRs showed consistent rates of most symptoms before and after applying Naranjo's algorithm, highlighting the stability of these adverse effects in relation to risperidone use. While some Extrapyramidal Symptoms (EPS), such as dystonia, was slightly more common in the standard-dose group (38.46%) compared to the low-dose group (23.68%), this difference was not statistically significant. Conversely, akathisia was reported more frequently in the low-dose group, suggesting that even lower doses can lead to EPS in certain patients. In case series of risperidone and EPS, all 10 cases developed EPS during treatment with risperidone, suggesting a clear association between risperidone and these adverse effects. Higher doses (6–8 mg) were linked to an earlier onset of EPS, whereas lower doses (1-4 mg) were associated with a delayed onset [29]. This dose-dependent pattern supports the hypothesis that higher doses of risperidone may increase the risk of EPS due to greater dopamine D2 receptor blockade, a mechanism well-documented in the literature [30, 31]. While several studies suggest that risperidone doses are associated with an increased risk of adverse effects, others have found no significant correlation between plasma concentrations of risperidone and these effects. This discrepancy may be due to the prominent role of 9-hydroxyrisperidone (9-OH-RIS), the main active metabolite from CYP2D6-catalyzed 9-hydroxylation, which is more relevant than risperidone itself in relation to ADRs [32].

Among central nervous system-related ADRs, insomnia was significantly more prevalent in the standard-dose group (23.08% *vs.* 5.26%, *p*= 0.03), reflecting the potential stimulating effects of higher risperidone doses. Risperidone's effects on sleep are thought to be mediated through its antagonistic action on serotonin (5-HT2) receptors. By blocking these receptors, risperidone may promote sleep by reducing serotonin activity, which can help regulate the sleep-wake cycle. This mechanism has the potential to improve sleep quality in patients with schizophrenia, who often experience sleep disturbances as part of their condition [33]. In a cross-sectional study evaluating the efficacy of olanzapine and risperidone for the treatment of paradoxical insomnia, 29 patients were randomly assigned to receive either 10 mg of olanzapine or 4 mg of risperidone daily over an 8-week period. The results indicated that 4 mg of risperidone was effective in improving sleep quality in patients with paradoxical insomnia, although olanzapine appeared to be more effective [34]. Thus, the higher prevalence of insomnia in the standard-dose group may be attributed to mechanisms other than serotonergic antagonism. The higher prevalence of insomnia in the standard-dose group is likely due to risperidone's relatively low affinity for H1 receptors compared to other atypical antipsychotics, resulting in less sedation [35].

Conversely, weight gain was more prominent in the low-dose group (68.42% *vs.* 42.31%, *p*= 0.04). This phenomenon can be explained by multiple contributing factors beyond dosage alone, including individual susceptibility, lifestyle behaviors, and pre-existing metabolic conditions [36]. Genetic predispositions, such as variations in metabolic enzyme activity (*e.g*., cytochrome P450 polymorphisms), can influence how patients metabolize risperidone [37], leading to differential risks for weight gain regardless of dose. Previous reviews of atypical antipsychotics have indicated that risperidone is associated with modest weight changes, which appear to be independent of the dose [38]. However, the study on the daily dose effects of risperidone on weight and other metabolic parameters found that for each 1 mg increase in dose, there was a corresponding weight increase of 0.16%, 0.29%, 0.21%, and 0.25% at 1,3, 6, and 12 months, respectively. These findings contrast with our results, which did not demonstrate a clear dose-dependent relationship between risperidone and weight change, suggesting potential variability in patient response or other influencing factors [39]. Our findings, which showed a higher proportion of weight gain, may be consistent with a meta-analysis that indicated the dose-response curve for risperidone plateaus at around 5 mg/day, with a maximum Mean Difference (MD) in weight gain of 1.82 kg [40].

This study has several strengths. The use of Naranjo's algorithm provided a structured and systematic assessment of Adverse Drug Reaction (ADR) causality, enhancing the reliability of the findings. Additionally, the study's design allowed for a detailed evaluation of ADR patterns across different risperidone dose groups, offering clinically relevant insights. However, certain limitations must be acknowledged. The relatively small sample size may restrict the generalizability of the results, particularly to broader populations. The lack of randomization introduces the possibility of selection bias, while the reliance on clinical records and self-reported data may lead to information bias. Furthermore, the cross-sectional nature of the study precludes conclusions about causal relationships between risperidone dose and ADRs.

Future research should utilize larger, longitudinal study designs with randomized approaches to establish clearer causal relationships. Additionally, factors such as gender, demographic characteristics, diagnostic variability, and the impact of co-medications should be further explored to provide a more comprehensive understanding of ADRs associated with risperidone.

The findings underscore the need for individualized dosing strategies, considering factors such as gender and susceptibility to specific ADRs. Clinicians should remain vigilant about the occurrence of EPS and central nervous system effects at standard doses, and metabolic monitoring is recommended even at lower doses. Adjusting doses based on patient-specific characteristics can optimize treatment outcomes while minimizing adverse effects.

## CONCLUSION

This study highlights dose-related differences in ADRs associated with risperidone in outpatients with schizophrenia. The findings suggest that lower doses of risperidone are linked to a higher incidence of weight gain, whereas standard doses are more strongly associated with insomnia.Other ADRs, such as dystonia and gastrointestinal symptoms, occurred at similar rates between the two dose groups without significant differences.

These findings emphasize the importance of personalized medication management, including careful dose titration, dose reduction when appropriate, and regular monitoring to enhance treatment safety and efficacy. Clinicians are encouraged to utilize standardized tools, such as the Naranjo Algorithm or patient-reported outcome measures, to systematically assess and address ADRs during follow-up visits. However, several limitations should be noted. The cross-sectional design restricts causal inferences, and the relatively small sample size may limit generalizability and the detection of rare ADRs. Potential biases from self-reported data and the lack of pharmacogenetic or pharmacokinetic assessments may also constrain the interpretation of results. Further studies with larger sample sizes and longitudinal cohort designs are needed to validate these findings and evaluate the long-term safety and dose-dependent effects of risperidone.

## STUDY LIMITATIONS

These findings emphasize the importance of personalized medication management, including careful dose titration, dose reduction when appropriate, and regular monitoring to enhance treatment safety and efficacy. Clinicians are encouraged to utilize standardized tools, such as the Naranjo Algorithm or patient-reported outcome measures, to systematically assess and address ADRs during follow-up visits. However, several limitations should be noted. The cross-sectional design restricts causal inferences, and the relatively small sample size may also limit generalizability and the detection of rare ADRs. Potential biases from self-reported data and the lack of pharmacogenetic or pharmacokinetic assessments may also constrain the interpretation of results. Further studies with larger sample sizes and longitudinal cohort designs are needed to validate these findings and evaluate the long-term safety and dose-dependent effects of risperidone.

## AUTHORS’ CONTRIBUTIONS

PK, SJ, and TB contributed to the design and implementation of the research, TB analyzed the results and wrote the manuscript, and WC conceived and supervised the project.

## Figures and Tables

**Table 1:** Baseline characteristics (*N*=64).

Characteristics	Low Dose Group (*N*=38)	Standard Dose Group (*N*=26)	*P*-value
**Gender**			
Female	24 (63.16)	9 (34.62)	0.02
Male	14 (36.84)	17 (65.38)	
**Age** (mean ± SD)	46.37±17.56	40.81±11.70	0.14
**Marital status**			
Single	21 (55.26)	20 (76.92)	0.08
Married	17 (44.74)	6 (23.08)	
**Treatment Duration** (months, mean ± SD)	28.57 ± 24.96	33.5 ± 34.49	0.51
**Diagnosis**			
F20.0 Paranoid schizophrenia	8 (21.05)	6 (23.08)	0.15
F20.9 Schizophrenia, unspecified	22 (57.89)	19 (73.08)	
F29.0 Unspecified nonorganic psychosis	8 (21.05)	1 (3.85)	
**Dose** (mg, median [min- max])	1.72 [1-3]	4.80 [4-6]	<0.01
**History of Drug allergy**			
No	36 (94.74)	24 (92.31)	0.69
Yes	2 (5.26)	2 (7.69)	

**Note:** *P*-values derived from the Chi-square test for categorical variables, Fisher’s Exact test where expected counts are less than 5, t-test, and Wilcoxon rank-sum test for continuous variables.

**Table 2:** Prevalence of adverse drug reactions.

Adverse Drug Reaction (ADR)	Initial Prevalence	Final Prevalence	Naranjo’s Score		
			**1-4**	**5-8**	**>8**
**Extrapyramidal symptoms**					
Dystonia	21 (32.81)	19(29.69)	11	8	0
Akathisia	6 (9.38)	6(9.38)	6	0	0
Parkinsonism	13 (20.31)	13(20.31)	11	2	0
Tardive dyskinesia	9 (14.06)	9(14.06)	8	1	0
**Central nervous system**					
Headache	7 (10.94)	7(10.94)	7	0	0
Insomnia	8 (12.5)	8(12.5)	7	1	0
Sedation	13 (20.31)	11(17.19)	4	7	0
Dry mouth	20 (31.25)	20(31.25)	12	8	0
Weight gain	37 (57.81)	37(57.81)	7	30	0
**Gastrointestinal system**					
Increase appetite	31 (48.44)	31(48.44)	17	14	0
Constipation	17 (26.56)	17(26.56)	0	17	0
**Endocrine and Reproductive system**					
Galactorrhea	1 (1.56)	1(1.56)	1	0	0
Irregular menstruation	10 (15.63)	10(15.63)	4	6	0
Loss of libido	3 (4.69)	3(4.69)	3	0	0
**Cardiovascular system**					
Orthostatic hypotension	11 (17.19)	11(17.19)	8	3	0
Palpitation	9 (14.06)	9(14.06)	5	4	0
**Others**					
Rash	2 (3.13)	2(3.13)	2	0	0
Nasal congestion	7 (10.94)	7(10.94)	4	3	0
**Total**	225	221			

**Table 3:** Comparison of adverse drug reactions between low and standard dose risperidone.

Adverse Drug Reaction ADR)	Low Dose Group (*N*=38)	Standard Dose Group (*N*=26)	*P*-value
**Extrapyramidal symptoms**			
Dystonia	9 (23.68)	10 (38.46)	0.20
Akathisia	5 (13.16)	1 (3.85)	0.39
Parkinsonism	8 (21.05)	5 (19.23)	0.86
Tardive dyskinesia	7 (18.42)	2 (7.69)	0.29
**Central nervous system**			
Headache	6 (15.79)	1 (3.85)	0.23
Insomnia	2 (5.26)	6 (23.08)	0.04
Sedation	7 (18.42)	4 (15.38)	1.00
Dry mouth	11 (28.95)	9 (34.62)	0.63
Weight gain	26 (68.42)	11 (42.31)	0.04
**Gastrointestinal system**			
Increase appetite	20 (52.63)	11 (42.31)	0.42
Constipation	13 (34.21)	4 (15.38)	0.15
**Endocrine and Reproductive system**			
Galactorrhea	1 (2.63)	0 (0.00)	1.00
Irregular menstruation	6 (15.79)	4 (15.38)	1.00
Loss of libido	1 (2.63)	2 (7.69)	0.56
**Cardiovascular system**			
Orthostatic hypotension	7 (18.42)	4 (15.38)	1.00
Palpitation	4 (10.53)	5 (19.23)	0.47
**Others**			
Rash	1 (2.63)	1 (3.85)	1.00
Nasal congestion	3 (7.89)	4 (15.38)	0.43

**Note:** *P*-values derived from the Chi-square test for categorical variables, Fisher’s Exact test where expected counts are less than 5.

## Data Availability

The data and supportive information are available within the article.

## LIST OF ABBREVIATIONS

ADRs: Adverse Drug Reactions
IOC: Index of Item Objective Congruence
IRB: Institutional Review Board
SD: Standard Deviation

## STANDARDS OF REPORTING

STROBE guidelines were followed.
